# Impact of Ultrasonic Time and Marinating Temperature on the Physicochemical Properties of Guinea Pig Meat (*Cavia porcellus*)

**DOI:** 10.3390/foods14223887

**Published:** 2025-11-13

**Authors:** Esteban Arteaga-Cabrera, Lina Escobar-Escobar, Oswaldo Osorio-Mora, Julián Andrés Gómez-Salazar

**Affiliations:** 1 Departamento de Alimentos, División de Ciencias de la Vida, Universidad de Guanajuato, Campus Irapuato-Salamanca, Ex-Hacienda El Copal, Carretera Irapuato-Silao km 9, Irapuato 36500, Guanajuato, Mexico; eh.arteagacabrera@ugto.mx (E.A.-C.); lp.escobarescobar@ugto.mx (L.E.-E.); 2 Grupo de Investigación GAIDA, Departamento de Procesos Industriales, Facultad de Ingeniería Agroindustrial, Universidad de Nariño, Pasto 52001, Colombia; osorio_oswaldo@udenar.edu.co

**Keywords:** ultrasound, water holding capacity, physicochemical properties, NaCl content, acetic acid content, quality properties of meat

## Abstract

Guinea pig (*Cavia porcellus*) meat is valued for its nutritional quality and ease of production. Marination is a key value-adding process, but it is limited by its long duration. Therefore, technologies that accelerate marination and improve tenderness are needed. The objective of this study was to evaluate the effect of ultrasound application time and temperature on the physicochemical parameters and yield of guinea pig meat during marination. The marination solution contained 1.9% NaCl, 1.9% acetic acid, and 0.51% oregano essential oil. Ultrasound treatment (200 W) was applied for 15–120 min, while static treatments were conducted at 20, 30, and 40 °C. Ultrasound-assisted marination of guinea pig meat improved NaCl uptake, reduced acetic acid content, and improved water-holding capacity. Moderate temperatures (40 °C) minimized weight loss, and short ultrasound times preserved color and texture. However, prolonged ultrasound exposure led to myofibrillar disruption and increased weight loss. Correlation analysis revealed that pH, influenced by NaCl and acetic acid, had a significant impact on moisture, texture, and water-holding capacity. Overall, the controlled application of ultrasound and temperature effectively optimized marination efficiency, enhanced functional properties, and preserved the quality of guinea pig meat.

## 1. Introduction

The guinea pig (*Cavia porcellus*) is a domesticated species that has been bred and consumed in the Andean region since at least the 15th century. In South American countries such as Peru, Ecuador, Bolivia, and Colombia, its production has historically been important as a food source and constitutes a traditional component of the diet in various rural communities [1].

The growing interest in guinea pig farming in South America is driven by multiple factors, including its adaptability to diverse environmental conditions, ease of management, low space and feed requirements, as well as its rapid growth and reproduction cycle [2]. These characteristics make the guinea pig a sustainable alternative to larger livestock species, which have higher production costs. Guinea pig meat is notable for its high protein content (21%) and low fat content (5%), surpassing conventional meats such as chicken or beef in nutritional quality [2,3], thereby reinforcing its value as a healthy and accessible high-quality protein.

Marination is a widely used technique to enhance the value of meat products by modifying their physicochemical and sensory properties through the incorporation of aqueous or oily solutions containing ingredients such as salt, phosphates, spices, and organic acids [4,5,6]. It can be performed by soaking, mixing, injection, or immersion to improve flavor, tenderness, color, juiciness, shelf life, and overall palatability [4]. Although traditionally applied to various meats, it also offers a promising approach to revalorize less common meats by improving their culinary attributes [7]. However, this process is relatively slow, and its efficiency depends on several factors such as the type of medium, the composition of the brine, solute concentration, and the application method [8]. Mechanical methods such as tumbling and injection have been employed to accelerate the diffusion of NaCl within the meat matrix; however, they may cause structural damage to the muscle tissue [9].

In response to these limitations, ultrasound-assisted marination has gained attention as an efficient, cost-effective, and eco-friendly alternative [10,11]. The technique relies on three main mechanisms: cavitation (formation, growth, and collapse of tiny bubbles in a liquid), mechanical vibration, and heat produced by ultrasound waves. These waves have frequencies above 20 kHz, beyond human hearing. Together, these mechanisms alter muscle microstructure, promote water and solute migration, and speed up mass transfer, such as NaCl (salt) penetration [8,12]. Ultrasonic cavitation effects that disrupt muscle fibers, cell membranes, and connective tissue, enhancing the action of the marination solution. These mechanical effects promote salt diffusion, increase ionic permeability, and improve water-holding capacity, tenderness, and flavor development. Consequently, ultrasound accelerates mass transfer processes such as NaCl penetration and contributes to superior marination efficiency [13,14,15].

Recent studies have demonstrated that the application of ultrasound in the marination of different types of meat significantly enhances curing efficiency, intensifies the process by reducing processing time, and promotes the incorporation of functional compounds, resulting in positive effects on texture and the sensory perception of the final product [16,17]. Temperature control during meat marination is crucial. It directly affects the quality, safety, and chemical and physical properties of the final product. Heating can cause proteins in muscle, connective tissue, and cell fluid of the meat to lose their natural shape. This creates structural changes that alter the texture and firmness of the meat [18,19,20]. Moreover, mild preheating or precooking treatments, such as marination at moderate temperatures, have been reported to enhance marinade diffusion and tenderness without reaching protein denaturation. Subsequent cooking or frying after such pre-treatments further determines the final texture, color, and microbial safety of the product [15,21]. Excessive heat can trigger chemical reactions that reduce the protective effect of natural enzymes. It can also release metals that speed up spoilage and break down fat-protein structures, making the fats more likely to go rancid [22]. In immersion marination, ingredients move into the meat by spreading throughout it. The temperature affects both the velocity and efficiency of spreading, which influences how evenly the marinade covers the meat and affects how much water the meat retains and its texture [23,24].

Although most previous studies have focused on evaluating the effects of cooking on the structure and quality of different types of meat, to the best of our knowledge, no research has yet examined the combined effect of ultrasound and temperature during marination on the physicochemical parameters of guinea pig (*Cavia porcellus*) meat. Therefore, this study aimed to evaluate the influence of ultrasonic treatment and temperature on the physicochemical characteristics of guinea pig meat during the marination process.

## 2. Materials and Methods

### 2.1. Materials

**Figure 1:**
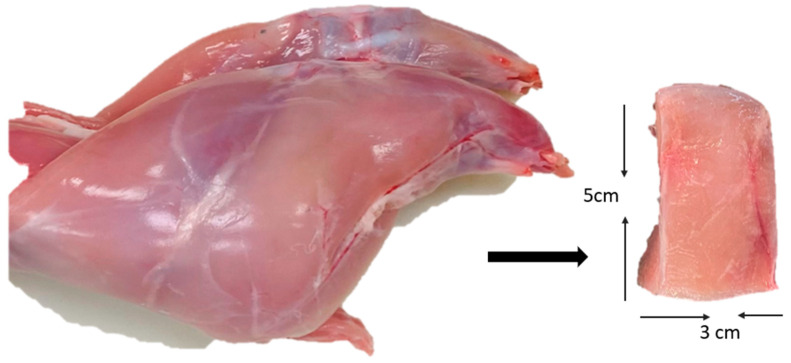
Guinea pig (*Cavia porcellus*) meat cuts.

Guinea pig (*Cavia porcellus*) carcasses were provided by the Department of Veterinary Medicine and Animal Science at the University of Guanajuato, in Irapuato, Guanajuato, Mexico. Legs and shoulders were used for the treatments. In the statistical analysis, individual animal variation was not considered due to the homogeneity in weight (950 g), age (90 days old), and sex (males). The samples were deboned and sliced into parallelepiped pieces measuring 50 mm in length, 30 mm in width, and 10 mm in thickness (Figure 1) an approximate weight of 20 ± 2 g. After cutting, the guinea pig samples were wrapped in plastic film and frozen at −20 ± 0.5 °C until marination experiments were conducted. Before treatment, the samples were thawed at 4 ± 0.5 °C.

Food-grade acetic acid (98% purity) was obtained from ILM Distribuciones S.A. de C.V. (Irapuato, Guanajuato, Mexico), while food-grade oregano essential oil (80% carvacrol) was supplied by Nature’s Blessing (Querétaro, Mexico). Food-grade sodium chloride (NaCl) from Fina (Mexico City, Mexico) was also used.

### 2.2. Experimental Conditions

The samples were marinated by immersion in solutions with NaCl concentrations of 1.9% (*v*/*v*), acetic acid 1.9% (*v*/*v*), and oregano essential oil 0.51%; these parameters were selected based on preliminary tests. All samples were immersed in the solutions (1:10 sample: solution). Static marinating was carried out at 20, 30, and 40 °C for 15, 30, 45, 60, 90, and 120 min. A Termolab static bath (model TE-B160MT, Termolab, Ciudad de Mexico, Mexico) was used to control the temperature [25]. The same procedure was carried out in samples immersed in an ultrasonic bath (Branson M2800, Danbury, CT, USA) at 40 kHz and 200 W of output power. The temperature of the ultrasonic bath was controlled and maintained at 25 °C, following the method described by Gómez-Salazar et al. [25]. The effective power output of the equipment was determined by calorimetry. A heating curve was established using 1000 g of distilled water, with the temperature recorded every 10 s until thermal equilibrium was reached [25].

### 2.3. Quality Attributes of Guinea Pig (Cavia porcellus) Meat

#### 2.3.1. NaCl Content

The NaCl content was determined using the Mohr method [25] with slight modifications. Briefly, 5 g of the sample were homogenized with 50 mL of distilled water using an Ultraturrax homogenizer (model T25, Janke & Kunkel, Staufen, Germany) for 5 min. The mixture was then centrifuged at 3500 rpm for 30 min at 20 °C, and 10 mL aliquots of the supernatant were titrated. Potassium dichromate (K_2_Cr_2_O_7_) was used as an indicator, and silver nitrate (AgNO_3_, 0.1 N) as the titrant. All titrations were performed in triplicate, and results were expressed as % NaCl.

#### 2.3.2. Acetic Acid Content

The acetic acid content was determined by titration with NaOH, following the method described by Goli et al. [26]. Briefly, 5 g of the sample were homogenized with 50 mL of distilled water using an Ultraturrax T25 homogenizer (Janke & Kunkel, Staufen im Breisgau, Germany) for 5 min. The homogenate was then centrifuged at 3500 rpm for 30 min at 20 °C, and 10 mL aliquots of the supernatant were titrated using phenolphthalein as an indicator. The titration was completed when a color change was observed.

#### 2.3.3. Moisture Content

The total moisture content of guinea pig meat after each treatment was determined according to the AOAC 950.46B method [25]. The evolution of the water content of each slice over time was determined by drying the sample to a constant weight at 103 ± 2 °C.

#### 2.3.4. Marination Weight Loss

Marination weight loss was determined by evaluating the difference in sample weight before and after marination [27]. Prior to weighing, surface water was carefully removed using absorbent paper towels to avoid excess liquid affecting the measurement. Each sample was gently blotted and weighed using an analytical balance with a precision of 0.01 g. The percentage (%) of weight loss was calculated using the initial and final weights of each sample. All measurements were performed in triplicate to ensure accuracy and reproducibility.

#### 2.3.5. Cooking Loss

After marination, the parallelepiped-shaped samples were weighed (*P_o_*), placed in plastic bags, and cooked in a water bath (TERMOLAB, model TE-B160MT) at a fixed temperature of 90 °C for approximately 5 min, or until the core temperature reached 70 °C. Samples were then cooled for 1 h, surface moisture was removed with paper towels, and they were reweighed (*P_c_*). The percentage of *Cooking Loss* was calculated using Equation (1) [27]:

(1) $$Cooking\;Loss\;\left(\%\right)=\;\frac{P_{o}-P_{c}}{P_{o}}\times \;100.$$

#### 2.3.6. Water Holding Capacity

The effect of the different marination treatments on the water holding capacity (*WHC*) was calculated as the variation in water content (Equation (2)) after immersion of the sample in the marination solution (*M*) relative to the initial water content (*M_o_*), according to the method described by Gómez-Salazar [5]:

(2) $$WHC=\;\frac{M-M_{O}}{M}$$

#### 2.3.7. Texture

Texture was determined using the puncture–penetration method on cooked samples. A Texture Analyzer (model TAX-T2, Stable Micro Systems) equipped with a flat cylindrical probe (2 mm, SMS P/2N) was used, operating at a fracture speed of 1 mm/s and a deformation level of 60% and 25 kg load cell on a heavy-duty platform at the test distance, 46 mm. The maximum peak of the force–time curve was recorded as hardness. Measurements were performed at five different points per sample [5].

#### 2.3.8. pH

For pH analysis, 5 g of the sample were homogenized with 50 mL of distilled water to obtain a uniform mixture. The electrode of a digital pH meter (model pH120, Conductronic), previously calibrated, was inserted into the homogenate under agitation. pH meter was calibrated with buffer 7.0 and 4.0. Measurements were carried out in triplicate.

#### 2.3.9. Color Determination

Color was measured in fresh meat and after each treatment. A colorimeter (Model Color Flex, Hunter Lab, Reston, VA, USA). The colorimeter was standardized using a cylindrical (black) light trap, followed by a standard white calibration plate. The CIE LAB system, Lightness (L*), green–red chromaticity (a*) and blue–yellow chromaticity (b*) was employed for the measurements [28]. The equipment was set with a D65 illuminant and a 10° standard observer. Color change was calculated using Equation (3):

(3) $$∆E=\sqrt{{\left(a_{0}-a\right)}^{2}+{\left(b_{0}-b\right)}^{2}{\left(L_{0}-L\right)}^{2},}$$

where the subscript “0” refers to the initial parameters.

### 2.4. Statistical Analysis

All measurements were performed in triplicate, and experimental data are presented as mean ± standard deviation. Statistical analyses were conducted using Statgraphics Centurion 19. Differences between means were evaluated by one-way analysis of variance (ANOVA), followed by a post hoc multiple comparison test using Tukey’s method, with a significance level of *p* < 0.05. Additionally, a Pearson correlation analysis was performed to evaluate the relationships among all measured response variables across all marination treatments of guinea pig (*Cavia porcellus*) meat. The analysis included physicochemical parameters (moisture, pH, water holding capacity, cooking loss, weight loss) and quality attributes (hardness and color parameters L*, a*, b*, and total color difference ΔE). All data from the response variables of all treatments were included in the analysis, allowing a comprehensive assessment of interrelationships among physicochemical and quality properties. Statistical significance was considered at *p* < 0.05, and high significance at *p* < 0.01. The correlation analysis was conducted using software Jamovi v2.4.11 (Jamovi, Sydney, Australia).

## 3. Results

### 3.1. Physicochemical Parameters of Guinea Pig (Cavia porcellus) Meat

#### 3.1.1. NaCl Content

**Figure 2:**
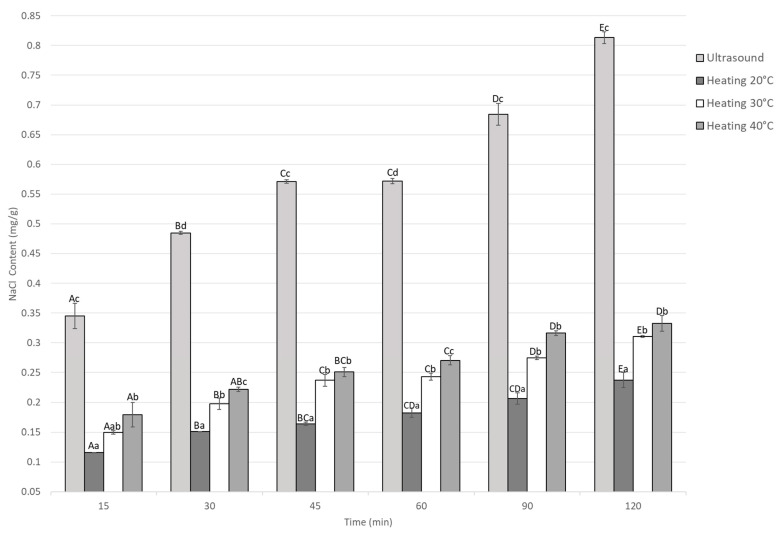
NaCl content of Guinea pig (*Cavia porcellus*) meat during marinating assisted by ultrasound or static heating. ^A–E^ Different letters indicate significant differences in time (*p* < 0.05). ^a–d^ Different letters indicate significant differences between treatments of the same time.

The NaCl content during marination under ultrasound and static heating conditions is shown in Figure 2. In all treatments, a significant increase in NaCl content was observed with increasing immersion time in the marinade (*p* < 0.05).

At each marinating time, the ultrasound-treated samples exhibited higher NaCl content than those marinated under thermal conditions (20, 30, and 40 °C). Increasing the temperature improved salt diffusion; however, ultrasound treatment consistently resulted in the highest NaCl content.

Within each treatment, NaCl content increased significantly (*p* < 0.05) with marination time, indicating continuous solute diffusion into the meat matrix. At the end of the marination process, the NaCl content in the treatments at 20, 30, and 40 °C was 0.23, 0.31, and 0.33 mg/g, respectively, for thermal marination. The ultrasound-assisted marination accelerated NaCl penetration, while heat-assisted treatments required longer times to achieve comparable levels. At 120 min, the ultrasound treatment reached the highest NaCl content (0.81 ± 0.01 mg/g), approximately three times greater than the group treated at 20 °C (0.23 ± 0.01 mg/g) and 1.5 times higher than the group treated at 40 °C (0.33 ± 0.01 mg/g). The interaction between time and treatment was significant (*p* < 0.05), indicating that both temperature and ultrasound intensity affected NaCl diffusion during marination.

#### 3.1.2. Acetic Acid Content

**Figure 3:**
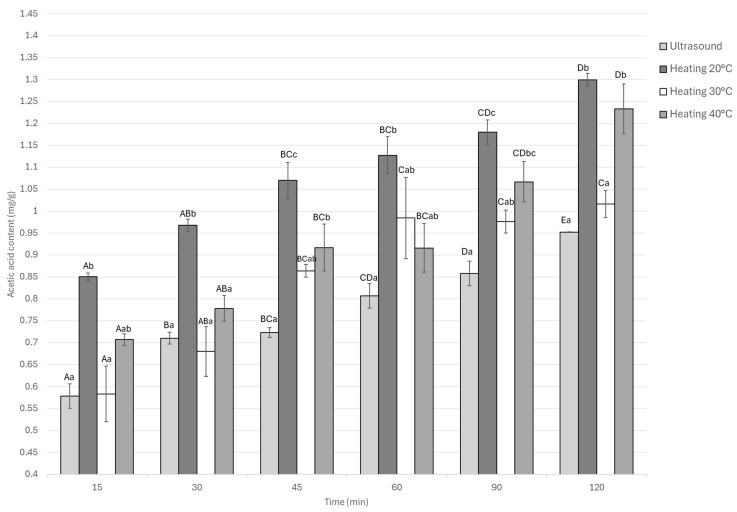
Acetic acid content of Guinea pig (*Cavia porcellus*) meat during marinating assisted by ultrasound or static heating. ^A–E^ Different letters indicate significant differences in time (*p* < 0.05). ^a–c^ Different letters indicate significant differences between treatments of the same time.

As shown in Figure 3, all treatments exhibited a progressive increase in acetic acid content as the marination time advanced. The static treatment at 20 °C displayed the highest acetic acid uptake throughout the process, reaching a maximum value of 1.29 mg/g. This was followed by the 40 °C treatment (1.23 mg/g), with no significant difference between them (*p* > 0.05). In contrast, the ultrasound-assisted treatment showed a slower increase, tending to stabilize after approximately 90 min of marination, and attained a final value of 0.95 mg/g, which was about 26% lower than the aforementioned thermal treatments.

This suggests that equilibrium was reached between 60 and 90 min under ultrasound, achieving substantial acid absorption without excessive accumulation that could affect sensory or textural attributes. The interaction between marination time and treatment was significant (*p* < 0.05), confirming that both temperature and sonication conditions influenced acid diffusion dynamics.

#### 3.1.3. Moisture Content

**Figure 4:**
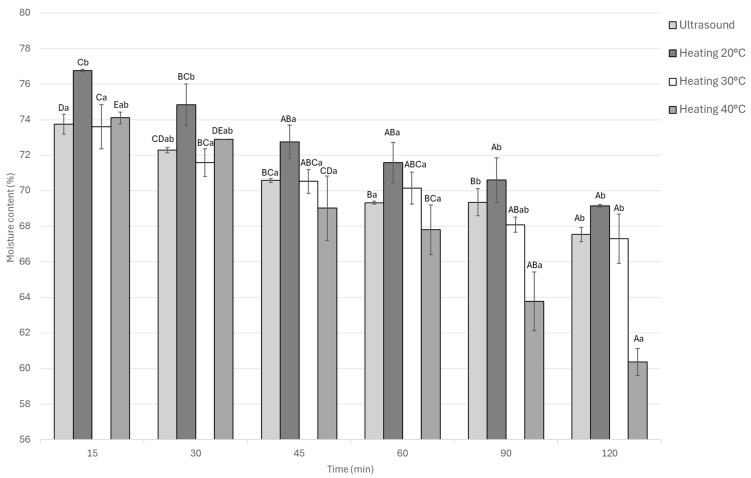
Moisture content of Guinea pig (*Cavia porcellus*) meat during marinating assisted by ultrasound or static heating. ^A–E^ Different letters indicate significant differences in time (*p* < 0.05). ^a,b^ Different letters indicate significant differences between treatments of the same time.

The moisture content of the samples decreased over time in all treatments. Significant differences (*p* < 0.05) were observed among treatments and marination times. At 15 min, heating at 20 °C showed the highest moisture content, as shown in Figure 4 is added to the preceding paragraph. In contrast, heating at 40 °C showed the lowest. This pattern continued throughout, with static marination at 20 °C retaining the most moisture. Both ultrasound and heating at 40 °C led to reduced moisture content. Within each treatment, the reduction in moisture content over time was statistically significant (*p* < 0.05). Compared across treatments, the total moisture decrease was approximately 6% for heating at 20 °C, while both ultrasound and heating at 30 °C led to an 8% decrease, and heating at 40 °C resulted in the largest decrease of about 8.5%.

#### 3.1.4. Marination Weight Loss

**Figure 5:**
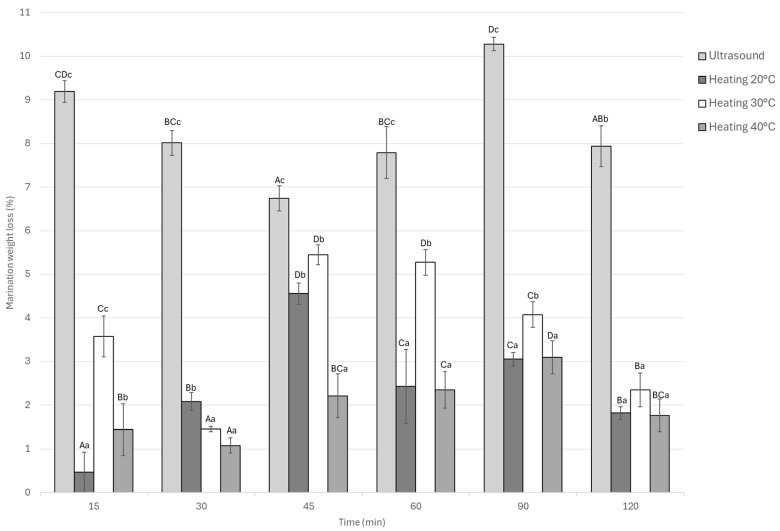
Marination weight loss of Guinea pig (*Cavia porcellus*) meat during marinating assisted by ultrasound or static heating. ^A–D^ Different letters indicate significant differences in time (*p* < 0.05). ^a–c^ Different letters indicate significant differences between treatments of the same time.

The statistical analysis of weight loss during the marination process revealed significant differences (*p* < 0.05) among treatments and across the evaluated times as seen in Figure 5. Overall, the ultrasound-assisted marination treatment exhibited the highest weight loss at all time points, reaching a maximum value of approximately 10.2% at 90 min, followed by a slight decrease toward 120 min. In contrast, static marination at 20 °C and 30 °C resulted in more moderate losses, stabilizing between 3% and 5%.

On the other hand, the ultrasound-assisted treatment exhibited intermediate behavior throughout the process, with weight loss values ranging from 2% to 5.2% over time. At the end of the marination period (120 min), the ultrasound treatment still exhibited the highest weight loss (7.9%), which was significantly different (*p* < 0.05) from the thermal marination treatments that showed lower final losses of 1.8%, 2.3%, and 1.7% for 20, 30, and 40 °C, respectively.

#### 3.1.5. Cooking Loss

**Figure 6:**
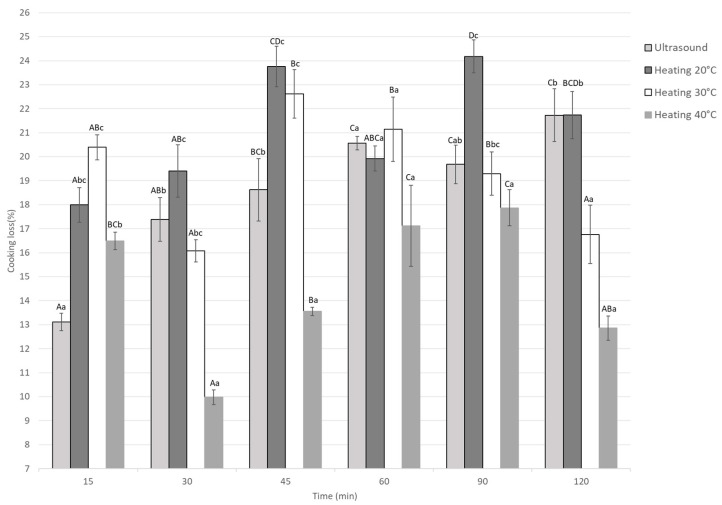
Cooking loss of Guinea pig (*Cavia porcellus*) meat during marinating assisted by ultrasound or static heating. ^A–D^ Different letters indicate significant differences in time (*p* < 0.05). ^a–c^ Different letters indicate significant differences between treatments of the same time.

Cooking weight loss in marinated guinea pig meat was significantly affected by treatment type and marination time (*p* < 0.05), as shown in Figure 6. Treatments at 20 °C and 30 °C showed higher weight losses, reaching up to 24.1% and 22.6%, especially at longer marination times (45–120 min). In contrast, heating at 40 °C produced the lowest losses for most times after cooking, with a minimum loss of 9.97% at 30 min. Ultrasound treatment also showed low and stable weight losses over time. At the same time points, statistical differences between treatments indicate that heating at 40 °C and ultrasound promote better water retention during cooking.

#### 3.1.6. Water Holding Capacity

**Figure 7:**
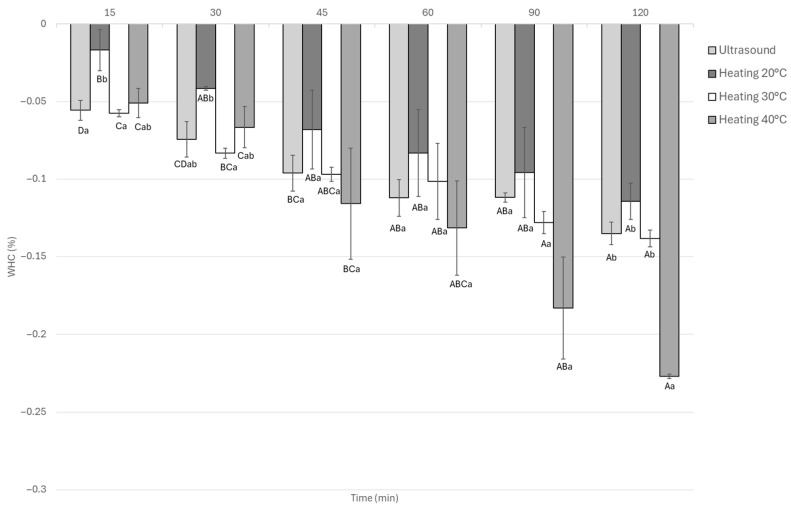
Water holding capacity Guinea pig (*Cavia porcellus*) meat during marinating assisted by ultrasound or static heating. ^A–D^ Different letters indicate significant differences in time (*p* < 0.05). ^a,b^ Different letters indicate significant differences between treatments of the same time.

Water-holding capacity (WHC) in marinated guinea pig meat was significantly (*p* < 0.05) affected by the type of treatment and marination time (Figure 7). Thermal treatments, particularly at 40 °C, led to greater water loss, reaching WHC values as low as 0.22% after 120 min. Heating at 20 °C gave the best short-term result, with minimal water loss (0.026%) at 15 min, but this effect did not last. In contrast, ultrasound treatment resulted in lower WHC values than 40 °C heating at all time points. There were no significant differences between 45 and 120 min, with a WHC of 0.22% indicating more consistent water retention during the process.

### 3.2. Effect of Treatments on Quality Properties of Guinea Pig (Cavia porcellus) Meat

The marination of guinea pig meat using ultrasound and thermal treatments significantly affected physicochemical and color parameters (*p* < 0.05), as shown in Table 1. The control group exhibited the highest pH (5.79), while the lowest value (3.76 at 90 min) occurred under ultrasound treatment. This reduction in pH suggests a higher diffusion rate of acidic components into the tissue. For thermal treatments, pH values stabilized after 60 min, maintaining an average of 4.2.

Regarding texture, thermal treatments, especially at 30 and 40 °C resulted in marked reductions in hardness, with minimum values of 0.32 N (40 °C, 30 min) and 0.65 ± 0.02 N (30 °C, 60 min), compared to the control (3.27 ± 0.11 N), suggesting enhanced muscle fiber degradation due to heat. However, ultrasound also significantly (*p* < 0.05) reduced hardness, reaching a minimum value of 0.74 N at 45 min, highlighting its potential to improve tenderness without thermal application.

Color parameters were also influenced by the treatments. The highest L* values (lightness) were obtained under ultrasound (up to 62.96 at 120 min), while a* values (redness) decreased significantly (*p* < 0.05) in ultrasound and 40 °C treatments, reaching as low as 0.64 in the ultrasound group. The b* parameter (yellowness) increased notably in ultrasound-treated samples, peaking at 13.01 after 45 min, whereas thermal treatments showed significant changes only after 45 min. The total color difference (Δ*E*) was significantly greater in ultrasound-treated samples (15.73 ± 1.41 at 120 min), indicating more pronounced surface modifications. In contrast, moderate temperature treatments (30 °C and 40 °C) produced subtler color variations, maintaining a visual appearance closer to fresh meat.

### 3.3. Correlation Analysis of Marinating Physicochemical Properties Guinea Pig (Cavia porcellus) Meat

**Figure 8:**
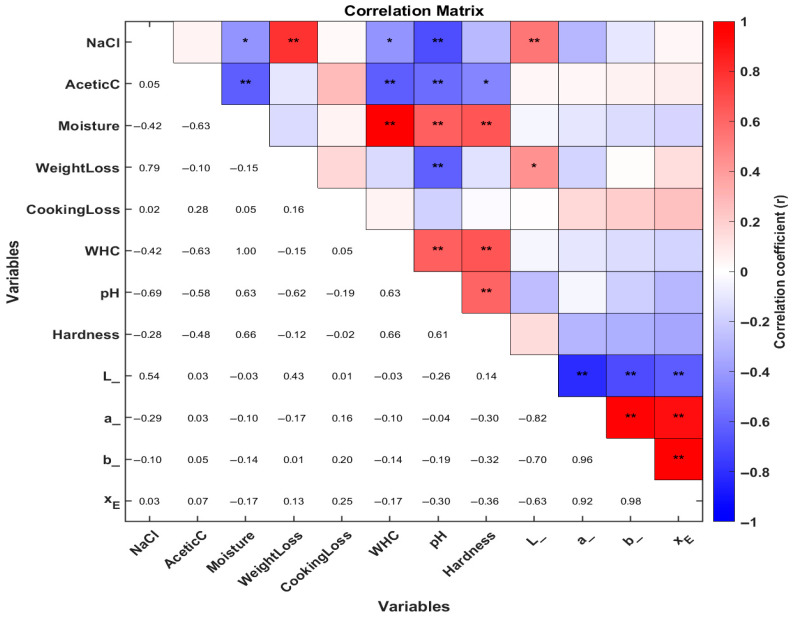
Heat map of Pearson’s correlation analysis between physicochemical and quality parameters under marination treatments of guinea pig (*Cavia porcellus*) meat. Red represents positive correlations, while blue indicates negative correlations. The asterisk (*) denotes the correlation coefficient, marked as significant (* *p* < 0.05, ** *p* < 0.01). L is L*, a is a*, b is b* and XE is ΔE

In Figure 8, the correlation analysis was performed to further evaluate the relationship between physicochemical changes, color changes, pH, and hardness of guinea pig meat across the different marination treatment groups. As shown in Figure 3, NaCl content exhibited a highly significant positive correlation with weight loss and the L* color parameter, whereas it showed a significant negative correlation with moisture and water-holding capacity (WHC), as well as a highly significant correlation with pH. In the case of acetic acid content, only negative correlations were observed, being significant with meat shear force and highly significant with moisture, WHC, and pH.

## 4. Discussion

According to the Codex Alimentarius (FAO/WHO) [29] Food-grade salt must contain at least 97% NaCl on a dry matter basis, excluding additives. In contrast, in meat products, the NaCl content is deliberately much lower (typically ≤3%) for health and formulation reasons, while ensuring microbial safety. In this study, the maximum NaCl content achieved by ultrasonic marination was 0.81 mg/g after 120 min, which is substantially below the traditional standard. This result shows that ultrasound-assisted marination can produce meat products with reduced sodium content. Such sodium reduction may be nutritionally desirable, as high salt intake is linked to hypertension and cardiovascular diseases [30]. Therefore, ultrasound-assisted marination offers a promising approach for developing healthier, lower-sodium meat products [30].

In all treatments, an increase in NaCl content was observed with marination time. In static marination, this increase is primarily driven by osmotic effects, where the NaCl gradient promotes solute diffusion into the muscle fibers [31,32]. Salt interacts with myofibrillar proteins, mainly myosin, to facilitate the penetration of the brine into the meat matrix. Furthermore, the positive effect of temperature on NaCl gain arises from enhanced diffusion coefficients and faster molecular mobility at elevated temperatures [27].

Temperature was also important, as it rose from 20 to 40 °C, NaCl uptake increased by about 30%, showing that heat boosts movement. In some studies of marinating, ultrasound and temperatures have been combined, and the joint effect of sound waves and heat led to the most salt absorption and the fastest balance [18]. This moderate heating can be considered a mild preheating step that enhances mass transfer without inducing strong protein denaturation. Similar pre-treatments have been reported in marinated meat systems, where mild thermal conditions facilitate solute diffusion [21].

After only 15 min, the ultrasonic treatment achieved more than twice the NaCl concentration of the static process, confirming the strong influence of acoustic cavitation on mass transfer efficiency. This phenomenon generates microjets and shock waves from the implosion of microbubbles near the tissue surface, forming microchannels that promote solute penetration [33]. Likewise, the pulsating activity of the bubbles induces a constant acoustic streaming that facilitates ion movement and enhances diffusion [17].

From a technological standpoint, these results highlight ultrasound as a promising tool to accelerate mass transfer and reduce processing times while maintaining a homogeneous distribution of salt [14]. From a nutritional and health perspective, the ability to obtain meat products with a lower total NaCl content is highly advantageous. Meat products commonly contain 10–28 mg NaCl/g of tissue at the end of processing, ref [5] in this study, the values were considerably lower, representing a significant advancement in the development of reduced-salt meat products.

Similar results were reported by Zhang et al. [34] in pork meat marinated at 20 kHz. The NaCl content increased significantly (*p* < 0.05) by 12% as the ultrasonic power rose from 100 to 700 W, and by 27% when comparing the conventional treatment with the 700 W ultrasound treatment. Likewise, in rabbit meat marination, it was reported that the maximum NaCl uptake increased by approximately 15% with the application of ultrasound compared to samples without ultrasound treatment [5]. Similarly, Zheng et al. [7] found that in beef marinated with tomato sour soup, ultrasound enhanced the penetration of salt and flavor compounds, reducing the total processing time by nearly 50%

At low temperatures, acid diffusion is better controlled, preventing early protein denaturation. Myofibrillar proteins keep their porous structure, supporting sustained acid absorption. At higher temperatures, partial protein coagulation reduces acid penetration [35].

Although ultrasound promotes mass transfer, it can also induce microcavitation and superficial denaturation. This forms a partial barrier that limits acid retention as marination progresses. Additionally, the mechanical effect of ultrasound may accelerate the release of intracellular fluid [36]. This reduces the capacity to retain acetic acid. In our results, higher acid transfer was observed in static treatments. The highest transfer was at 20 °C, while the lowest occurred under ultrasound treatment. This may be explained by the effects of cavitation, which impacts both molecular interactions in the marinade solution and the meat tissue, decreasing attractive forces and weakening the molecules [37]. This could explain the lower acetic acid levels in ultrasound-treated samples compared with static ones.

The opposite behavior observed for NaCl gain is attributed to the higher solubility of organic acids in water and the drag effect generated during mass transfer. As the temperature increases, the kinetic energy of water molecules rises. This allows them to overcome the intermolecular forces of acetic acid and helps with its dissolution [38]. The limited effect of ultrasound on acetic acid gain is related to the rapid series of alternating compressions and expansions. This process induces dehydration and promotes moisture migration toward the surface in the meat tissue [39].

Compared to other studies, the final acetic acid levels observed in this work (0.8–1.3 mg/g) are lower than those reported for marination with commercial vinegar or strong organic acids (1.5–2.0 mg/g) in pork [34]. However, the application of ultrasound allows for a reduced acid concentration in the formulation while achieving equivalent effects in a shorter time and with a lower sensory impact, representing a technological and food safety advantage.

Acidic marination is a common practice for tough meats to improve tenderness. Acidic marinades lower meat pH, enhancing tenderness through collagen and myofibrillar protein solubilization and muscle swelling [40]. Previous studies have also shown the effect of acidic marinades on the texture of the meat. Gómez-Salazar et al. (2018) observed an opposite effect with marination of rabbit meat using 1.5% citric acid, where meat hardness increased [5].

In a study evaluating the effect of acetic acid on marinated Little Tunny fillets, the optimal concentration was found to be 1.1% of the marinade solution, achieving favorable results for weight loss, texture, and sensory acceptance [40]. In our study, ultrasound-assisted marination achieved lower acid levels, while static treatment reached similar values after 60 min.

Moisture reduction during marination is mainly governed by osmotic and diffusion-driven mechanisms, which are influenced by both temperature and ultrasound application. At higher temperatures, the increased kinetic energy of water molecules and the partial disruption of cell membranes enhance the migration of water toward the surface [18]. The ultrasound-assisted treatment accelerated water loss, particularly during the first 60 min, suggesting an intensifying effect of ultrasound on mass transfer. This phenomenon is related to acoustic cavitation, which generates microchannels within the tissue, enhancing solute diffusion and promoting water migration to the surface [33].

These physical effects explain the greater dehydration observed under ultrasound and elevated temperature conditions, as reported previously for marinated rabbit and poultry meats, where ultrasound promoted faster moisture transfer without severely compromising tissue integrity [5,7,34]. Furthermore, the greater moisture loss observed at 40 °C may result from the heating medium and thermal stress of muscle cells, which facilitates the flow of free water toward the surface.

However, excessive water loss can compromise the juiciness of the final product, highlighting the importance of balancing both temperature and ultrasound exposure time. Overall, the results suggest that ultrasound-assisted marination represents an effective strategy to enhance process efficiency without relying on prolonged durations or high temperatures, thereby optimizing the functional quality of the product while maintaining its moisture [4,41].

Ultrasound-assisted marination promoted greater water loss compared to static heating treatments. This effect is associated with acoustic cavitation, which generates microchannels in the muscle tissue and enhances mass transfer, facilitating the migration of water and solutes. However, the lowest weight loss observed in the sample marinated at 20 °C suggests that marination efficiency can be improved without causing excessive tissue dehydration. This indicates that temperature is the most critical factor affecting weight loss during marination, while ultrasound acts as a process enhancer. Additionally, prolonged ultrasound treatments may damage muscle tissue, disrupting the myofibrillar structure and facilitating greater water and marinade solution exchange [42]. Overall, weight loss during marination depends on the treatment conditions and the type of meat used, with reported values ranging from 2% to 12%. The results of this study fall within this range, confirming consistency with the literature [5,42,43].

Cooking losses are directly related to the water-holding capacity of meat, which depends on the structural integrity and functional state of myofibrillar proteins. When marination involves low NaCl concentrations combined with an acidic medium, electrostatic repulsion between proteins increases, facilitating water release during cooking [9]. Additionally, approximately 85% of the water in meat, immobilized or trapped by myofibrillar proteins, can be easily released if the protein structure is altered or denatured by acid [44]. Additionally, NaCl can solubilize myofibrillar proteins that act as internal emulsifiers, thereby influencing the retention of water within the tissue [37].

In this study, ultrasound treatment also influenced cooking losses. Lower losses occurred with short ultrasound durations, with a significant reduction only in samples treated for 15 min. This suggests the applied power and acidic marination did not damage muscle tissue. Previous reports show pressures and microjets from cavitation can disrupt muscle and release more water [37]. Ultrasound for 15 min lets meat retain more water, improving juiciness. Therefore, short ultrasound treatment leads to moderate weight loss and effective water retention without excessive dehydration.

This result aligns with previous studies that report cooking losses in ultrasound-marinated beef range between 40% and 45%, indicating that ultrasound treatment significantly improves water retention [7]. Similarly, ultrasound-marinated pork showed a reduction in cooking loss from approximately 35% to 24%, which also suggests that ultrasound enhances water-holding capacity during cooking [24].

The comparison between treatments at each time point showed statistically significant differences (*p* < 0.05), with ultrasound being one of the treatments with the best performance in terms of water-holding capacity (WHC). Thermal treatments, particularly at 40 °C, exhibited a progressive decrease in WHC as marination time increased, likely associated with protein denaturation and the disruption of the myofibrillar matrix caused by prolonged heating. In contrast, ultrasound-assisted samples exhibited superior and more stable WHC throughout marination, especially during the first 60 min, suggesting that ultrasound exerts a protective or modulatory effect on muscle proteins. This behavior indicates that elevated thermal conditions deteriorate the protein structures responsible for water retention, promoting exudation, while ultrasound helps maintain structural integrity. Similar effects have been reported in rabbit meat, where ultrasound reduced WHC loss during marination [13].

The enhanced WHC observed under ultrasound may be attributed to the mild mechanical action of acoustic cavitation, which generates microchannels and increases tissue porosity without damaging the myofilament network. These structural modifications improve the diffusion of salt and organic acids while maintaining the proteins responsible for water retention [11,45]. Similarly, Alarcon-Rojo et al. [46] demonstrated that the use of low-intensity ultrasound helps preserve the functional properties of muscle proteins during marination. Ultrasound-assisted marination in rabbit meat showed WHC values close to 0.120 [5], which are comparable to those observed in this study using guinea pig meat.

pH is a critical indicator of meat quality, as it influences enzymatic activity, tenderness, and microbial stability. The observed pH decline under ultrasound and thermal treatments reflects enhanced diffusion of acetic acid and NaCl from the marinade. Ultrasound promoted a greater decrease, likely due to cavitation, which enhances mass transfer and disrupts cell membranes. However, the values obtained in this study were lower than those reported in previous research. Zheng et al. [7] observed pH values ranging from 5.84 to 4.87 in ultrasound-marinated beef, while [47] reported an initial pH of 6.03 in rabbit meat, which decreased to 5.74 after ultrasound treatment. Similarly, Gómez et al. [5] found pH values as low as 4.97 in rabbit meat marinated with NaCl and acetic acid solutions.

Such acidic conditions favor the activation of cathepsins (pH 3.5–5.0), enhancing proteolysis and collagen solubilization, thus improving tenderness [48]. In this context, ultrasound appeared to achieve a sufficiently low pH to optimize cathepsin proteolytic activity, resulting in greater degradation of myofibrillar proteins and solubilization of connective tissues. Consequently, an improvement in meat tenderness was observed, evidenced by a significant reduction in muscle fiber diameter and thickness, consistent with previous reports [49,50].

One of the main benefits of marination on meat quality is the improvement of tenderness, as chewiness and consumer acceptability are directly related to this parameter [51]. The findings of this study are consistent with those reported by [48], who demonstrated that acidic marination significantly (*p* < 0.05) affected pork tenderness, reducing shear force from 44.33 N to 20.76 N, resulting in a 50% softer texture. Similarly, other studies have shown through sensory and instrumental analyses that marination improves tenderness in beef, poultry, and pork [26,42,51].

Meat tenderization is primarily associated with protein structure disruption caused by proteolysis. During marination, NaCl and acetic acid affect the electrostatic interactions between protein and myofibrillar chains, altering the structure of the endomysium and perimysium within the connective tissue, thereby weakening them and directly influencing meat texture [26,48]. Ultrasound treatments of sufficient intensity can disrupt lysosomes and release endogenous proteolytic enzymes [37] that act on muscle tissue. The vibration generated by oscillating ultrasonic waves weakens the intermolecular forces among proteins, leading to muscle softening and facilitating the release of lysosomal Ca^2^-dependent cathepsins, thus improving tenderness [36]. This agrees with [42], who reported a reduction in shear force from 9 to 6 N in chicken breast after 30 min of ultrasound-assisted marination. Additionally, the presence of NaCl and acetic acid further affects muscle proteins, weakening the fiber structure and reducing the force required to disrupt the meat matrix [52]. This effect increases with sonication time due to the expansion of myofibrils, enhancing the penetration of the marination solution and promoting greater interaction between the marinade and muscle tissue [37].

Myoglobin, a key protein influencing meat color, can be affected by oxidation reactions with salts and acids, and even through auto-oxidation under environmental or storage conditions [52]. Acetic acid directly impacts meat by lowering the pH, promoting exposure of the heme group, and inducing partial loss of the helical structure, resulting in greater susceptibility of myoglobin to denaturation [53]. Meat color can also be influenced by pH, cooking, and preservation processes, but the most critical factor is the oxidation state of myoglobin [54]. In meat matrices, it is desirable that total color difference (ΔE) values remain below 5, since consumers strongly associate color with freshness, nutritional value, texture, and flavor [27]. Acidic marinades have been reported to cause noticeable color changes; for instance, Gómez-Salazar et al. [5] observed ΔE values ranging from 16 to 32 as NaCl and citric acid concentrations increased, which was attributed to lipid and protein oxidation.

In the present study, a decrease in redness (a*), this phenomenon can be attributed to myoglobin denaturation and oxidation reactions induced by external factors, leading to paler colors with lower red pigmentation [53].

The NaCl and acetic acid contents showed a positive correlation with weight loss during marination, indicating that higher concentrations of NaCl and acid promote greater water and solute loss due to osmotic effects. This trend was also reported by Zhang [17], who found that the acidity and salinity of the marination medium directly influence of water and solutes during treatment.

Hardness and color parameters were closely linked. Shear force showed a positive correlation with moisture and water-holding capacity (WHC), meaning meat with more water is more tender. The L* value (lightness) was positively correlated with NaCl content and weight loss. In contrast, a* (red) and b* (yellow) correlated only with L*. These findings align with Zhang et al. [7] who found that marination-induced structural changes affected both appearance and sensory qualities of meat.

## 5. Conclusions

Ultrasound-assisted marination increased NaCl uptake in guinea pig meat versus static marination, regardless of temperature. Ultrasound also reduced acetic acid content compared to heat-assisted marination. Both temperature and ultrasound significantly (*p* < 0.05) affected marination efficiency and meat water-holding capacity. Short ultrasound treatments (15 min) improved marination solution penetration without causing excessive muscle damage, thus maintaining juiciness and reducing cooking water loss. In contrast, prolonged ultrasound led to more myofibrillar disruption and water release, while lower temperatures (20–30 °C) increased weight loss due to incomplete protein denaturation. These results demonstrate that controlled ultrasound and low temperatures maximize marination efficiency, enhance water retention, and preserve sensory quality. Ultrasound during marination also affected color, decreasing redness (a*) and increasing yellowness (b*). Correlation analysis showed that pH was highly sensitive to NaCl and acetic acid concentrations, which in turn influenced moisture, weight loss, water-holding capacity, and texture (hardness). Future research could build on these findings by exploring the effects of cooking or frying on the sensory and commercial attributes of marinated meat. Furthermore, evaluating the impact of marination on lipid oxidation, digestibility, and the nutritional and functional properties would deepen understanding of its potential applications in guinea pig meat processing and contribute to the advancement of sustainable meat technologies.

## Figures and Tables

**Table 1:** Effects of marinating treatments on the quality properties of Guinea pig (*Cavia porcellus*) meat.

		pH	Hardness (N)	L*	a*	b*	ΔE
Control		5.79 ± 0.07 ^a^	3.27 ± 0.11 ^a^	47.83 ± 1.02 ^a^	5.14 ± 0.56 ^a^	6.97 ± 0.47 ^e^	0 ± 0
US	15	4.48 ± 0.04 ^b^	2.75 ± 0.11 ^b^	52.62 ± 0.74 ^b^	2.8 ± 0.2 ^b^	11.63 ± 0.02 ^b^	7.09 ± 0.58 ^c^
	30	4.05 ± 0.04 ^d^	1.12 ± 0.06 ^c^	59.34 ± 0.29 ^c^	3.29 ± 0.16 ^b^	10.23 ± 0.15 ^c^	12.11 ± 0.33 ^b^
	45	4.23 ± 0.04 ^c^	0.74 ± 0.02 ^d^	50.62 ± 1.12 ^ab^	2.97 ± 0.32 ^b^	13.01 ± 0.68 ^a^	7.06 ± 0.76 ^c^
	60	3.94 ± 0.05 ^e^	0.76 ± 0.04 ^d^	58.2 ± 0.68 ^c^	2.94 ± 0.07 ^b^	10.4 ± 0.1 ^c^	11.14 ± 0.65 ^b^
	90	3.76 ± 0.01 ^e^	0.76 ± 0.07 ^d^	60.8 ± 2.31 ^cd^	0.64 ± 0.07 ^c^	9.04 ± 0.14 ^d^	13.9 ± 2.18 ^ab^
	120	3.91 ± 0.01 ^f^	0.8 ± 0.04 ^d^	62.96 ± 1.47 ^d^	1.32 ± 0.08 ^c^	8.92 ± 0.06 ^d^	15.73 ± 1.41 ^a^
		pH	Hardness (N)	L*	a*	b*	ΔE
Control		5.79 ± 0.07 ^a^	3.27 ± 0.11 ^a^	47.83 ± 1.02 ^c^	5.14 ± 0.56 ^ab^	6.97 ± 0.47 ^a^	0 ± 0
20 °C	15	4.78 ± 0.03 ^b^	3.31 ± 0.05 ^a^	44.91 ± 0.48 ^b^	6.47 ± 0.16 ^b^	7.38 ± 0.09 ^ab^	3.24 ± 0.58 ^a^
	30	4.55 ± 0.05 ^b^	1.18 ± 0.06 ^bc^	41.4 ± 0.16 ^a^	4.43 ± 0.23 ^a^	8.66 ± 0.31 ^bc^	6.69 ± 0.33 ^c^
	45	4.43 ± 0.02 ^c^	0.77 ± 0.05 ^e^	53.39 ± 0.39 ^d^	4.59 ± 0.84 ^a^	8.32 ± 0.16 ^abc^	7.79 ± 0.75 ^bc^
	60	4.28 ± 0.03 ^d^	1.05 ± 0.02 ^cd^	55.30 ± 0.51 ^d^	3.61 ± 0.09 ^a^	11.07 ± 0.38 ^e^	8.67 ± 0.65 ^d^
	90	4.17 ± 0.03 ^d^	1.33 ± 0.07 ^b^	49.79 ± 2.31 ^c^	4.50 ± 1.10 ^a^	10.37 ± 1.26 ^de^	4.55 ± 2.18 ^ab^
	120	4.21 ± 0.01 ^d^	0.88 ± 0.04 ^de^	54.18 ± 0.49 ^d^	3.63 ± 0.06 ^a^	9.53 ± 0.09 ^cd^	7.01 ± 1.41 ^c^
		pH	Hardness (N)	L*	a*	b*	ΔE
Control		5.79 ± 0.07 ^a^	3.27 ± 0.11 ^a^	47.83 ± 1.02 ^a^	5.14 ± 0.56 ^a^	6.97 ± 0.47 ^ab^	0 ± 0
30 °C	15	4.78 ± 0.03 ^b^	3.31 ± 0.05 ^a^	48.76 ± 1.06 ^a^	6.77 ± 1.23 ^ab^	7.81 ± 1.38 ^a^	2.55 ± 1.03 ^a^
	30	4.55 ± 0.05 ^b^	1.18 ± 0.06 ^b^	37.93 ± 0.08 ^b^	5.09 ± 0.12 ^abc^	6.11 ± 0.16 ^b^	7.05 ± 0.19 ^d^
	45	4.43 ± 0.02 ^c^	0.77 ± 0.05 ^c^	21.09 ± 0.12 ^c^	4.53 ± 0.12 ^abc^	5.52 ± 0.51 ^b^	6.62 ± 0.44 ^cd^
	60	4.28 ± 0.03 ^d^	0.65 ± 0.02 ^c^	19.23 ± 0.59 ^c^	3.16 ± 0.05 ^bc^	3.47 ± 0.51 ^c^	5.46 ± 0.50 ^bc^
	90	4.17 ± 0.03 ^d^	0.83 ± 0.07 ^bc^	16.41 ± 1.22 ^c^	3.11 ± 0.08 ^bc^	3.26 ± 0.26 ^c^	5.01 ± 0.03 ^b^
	120	4.21 ± 0.01 ^d^	0.88 ± 0.04 ^bc^	17.14 ± 0.07 ^c^	3.35 ± 0.53 ^bc^	2.07 ± 0.18 ^c^	6.63 ± 0.01 ^cd^
		pH	Hardness (N)	L*	a*	b*	ΔE
Control		5.79 ± 0.07 ^a^	3.27 ± 0.11 ^a^	47.83 ± 1.02 ^a^	5.14 ± 0.56 ^a^	6.97 ± 0.47 ^ab^	0 ± 0
40 °C	15	4.82 ± 0.04 ^b^	1.33 ± 0.09 ^b^	44.43 ± 0.68 ^ab^	6.06 ± 0.25 ^cd^	6.39 ± 0.17 ^b^	3.57 ± 0.72 ^bc^
	30	4.59 ± 0.01 ^c^	0.32 ± 0.02 ^c^	46.05 ± 0.99 ^ab^	6.44 ± 0.27 ^d^	6.95 ± 0.22 ^b^	2.25 ± 0.90 ^abc^
	45	4.52 ± 0.01 ^c^	0.40 ± 0.02 ^c^	46.63 ± 1.90 ^abc^	4.54 ± 0.51 ^b^	6.99 ± 0.80 ^b^	2.16 ± 0.46 ^ab^
	60	4.34 ± 0.02 ^d^	0.57 ± 0.04 ^c^	47.21 ± 0.66 ^abc^	6.56 ± 0.18 ^d^	7.59 ± 0.29 ^bc^	1.77 ± 0.05 ^a^
	90	4.28 ± 0.03 ^d^	0.55 ± 0.04 ^c^	45.54 ± 0.67 ^ab^	3.31 ± 0.27 ^a^	4.39 ± 0.30 ^a^	3.94 ± 0.39 ^c^
	120	4.27 ± 0.02 ^d^	0.48 ± 0.04 ^c^	49.11 ± 1.7 ^c^	6.86 ± 0.15 ^d^	8.61 ± 0.57 ^c^	2.80 ± 0.82 ^abc^

Note: ^a–f^ means within the same column with different letters differ significantly between treatments (*p* < 0.05), Control: time 0 min.

## Data Availability

The original contributions presented in this study are included in the article. Further inquiries can be directed to the corresponding authors.
